# HVEM structures and mutants reveal distinct functions of binding to LIGHT and BTLA/CD160

**DOI:** 10.1084/jem.20211112

**Published:** 2021-10-28

**Authors:** Weifeng Liu, Ting-Fang Chou, Sarah C. Garrett-Thomson, Goo-Young Seo, Elena Fedorov, Udupi A. Ramagopal, Jeffrey B. Bonanno, Qingyang Wang, Kenneth Kim, Scott J. Garforth, Kiyokazu Kakugawa, Hilde Cheroutre, Mitchell Kronenberg, Steven C. Almo

**Affiliations:** 1 Department of Biochemistry, Albert Einstein College of Medicine, Bronx, NY; 2 La Jolla Institute for Immunology, La Jolla, CA; 3 Laboratory for Immune Crosstalk, RIKEN Center for Integrative Medical Sciences, Yokohama, Japan; 4 Division of Biological Sciences, University of California San Diego, La Jolla, CA; 5 Department of Physiology and Biophysics, Albert Einstein College of Medicine, Bronx, NY

## Abstract

HVEM is a TNF (tumor necrosis factor) receptor contributing to a broad range of immune functions involving diverse cell types. It interacts with a TNF ligand, LIGHT, and immunoglobulin (Ig) superfamily members BTLA and CD160. Assessing the functional impact of HVEM binding to specific ligands in different settings has been complicated by the multiple interactions of HVEM and HVEM binding partners. To dissect the molecular basis for multiple functions, we determined crystal structures that reveal the distinct HVEM surfaces that engage LIGHT or BTLA/CD160, including the human HVEM–LIGHT–CD160 ternary complex, with HVEM interacting simultaneously with both binding partners. Based on these structures, we generated mouse HVEM mutants that selectively recognized either the TNF or Ig ligands in vitro. Knockin mice expressing these muteins maintain expression of all the proteins in the HVEM network, yet they demonstrate selective functions for LIGHT in the clearance of bacteria in the intestine and for the Ig ligands in the amelioration of liver inflammation.

## Teaser

HVEM is a TNF family receptor that binds a TNF protein and Ig family proteins. Using structural biology and mutagenesis, we identified HVEM muteins with selective ligand binding. We verified their function in vivo in models of inflammation and infection.

## Graphical Abstract

**Figure:**
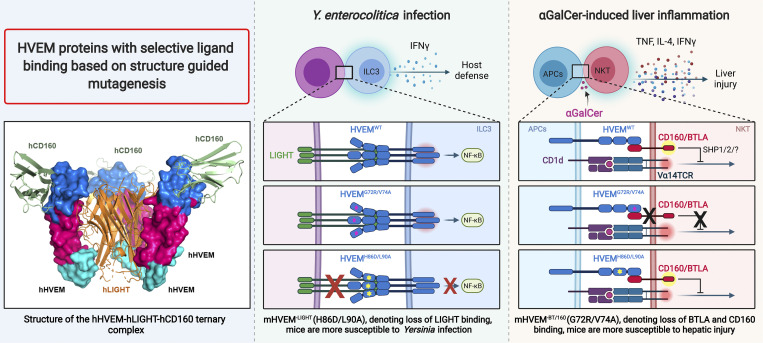

## Introduction

Members of the TNF receptor (TNFR) superfamily (TNFRSF) regulate diverse processes, but in several cases, understanding these processes is hampered by the ability of receptors and ligands to bind to multiple partners (Bossen et al., 2006). One prominent example is provided by the herpes virus entry mediator (HVEM), or TNFRSF14, initially identified as important for entry of HSV through recognition of HSV glycoprotein D (Montgomery et al., 1996; Whitbeck et al., 1997). Subsequently, a TNF superfamily (TNFSF) ligand for HVEM was characterized, known as LIGHT (homologous to lymphotoxin, exhibits inducible expression and competes with HSV glycoprotein D for binding to herpesvirus entry mediator, a receptor expressed on T lymphocytes) or TNFSF14 (Harrop et al., 1998a; Harrop et al., 1998b). Engagement of HVEM by LIGHT is implicated in multiple responses. For example, in T lymphocytes, it stimulates proliferation, cytokine production, and the development of CD8 T cell memory (Desai et al., 2017; Harrop et al., 1998a; Harrop et al., 1998b; Tamada et al., 2000). LIGHT also engages HVEM to stimulate cytokine production by type 3 innate lymphoid cells (ILC3s; Seo et al., 2018), and in keratinocytes, it binds HVEM to stimulate periostin, contributing to atopic dermatitis (Herro et al., 2018).

LIGHT also binds to another TNFRSF member, lymphotoxin-β receptor (LTβR or TNFRSF3), which is expressed by stromal and myeloid lineages. This interaction regulates lymph node formation, dendritic cell migration (Zhu et al., 2011), and IL-12 production by dendritic cells (Okwor et al., 2015). The LIGHT-LTβR interaction also has been reported to induce apoptosis of cancer cells (Zhai et al., 1998), it is important for macrophage activity in wound healing (Petreaca et al., 2012), and it influences lipid metabolism by regulating hepatic lipase expression in hepatocytes (Chellan et al., 2013; Lo et al., 2007). Furthermore, LIGHT participates in additional processes in which a specific receptor has not been implicated, including the resolution of inflammation in an experimental autoimmune encephalomyelitis (Mana et al., 2013), the induction of adipocyte differentiation (Tiller et al., 2011), and the induction of osteoclastogenic signals (Brunetti et al., 2014; Hemingway et al., 2013).

HVEM also binds Ig superfamily (IgSF) molecules B and T lymphocyte attenuator (BTLA or CD272) and CD160. HVEM engages in bidirectional signaling, serving not only as a receptor but also as a ligand for IgSF receptor signaling (Steinberg et al., 2011). HVEM–BTLA engagement delivers an overall inhibitory immune response (Murphy and Murphy, 2010), while the interaction between HVEM and CD160 on T cells can either attenuate the activities of specific subsets of CD4 T lymphocytes or enhance the activity of CD8 T cells (Cai et al., 2008; Tan et al., 2018). Notably, engagement of CD160 by HVEM also controls cytokine production by natural killer cells and is important for mucosal immunity (Shui et al., 2012; Tu et al., 2015; Whitbeck et al., 1997). Furthermore, HVEM was reported to interact with synaptic adhesion-like molecule 5, mainly expressed in brain, to confer immune privilege in the central nervous system (Zhu et al., 2016). CD160 also binds to some MHC class I molecules (Le Bouteiller et al., 2002; Maeda et al., 2005), further expanding the complexity of this protein–protein interaction network.

The promiscuous interactions of HVEM pose challenges for characterizing the mechanistic contributions of HVEM-associated pathways in different immune responses and diseases. Conditional knockouts can isolate effects in particular cell types, but elimination of expression of one protein, for example LIGHT, not only abolishes LIGHT–HVEM binding but also eliminates LIGHT–LTβR binding and may also indirectly affect HVEM interactions with its IgSF ligands by altering the availability of HVEM (Steinberg et al., 2011). This complexity may make it difficult to reach definitive conclusions about the relevant binding partners responsible for a phenotype, and it may account for circumstances in which the phenotypes in whole-body receptor and corresponding ligand knockouts did not agree (Giles et al., 2018). Herein, in order to better understand this receptor–ligand network, we set out to test mutants of HVEM with selective ligand binding. Based on multiple crystal structures, including the human HVEM (hHVEM)–LIGHT–CD160 ternary complex, we performed extensive epitope mapping and engineering of selective mouse HVEM (denoted as mHVEM) mutants. HVEM muteins were expressed in mice to show definitively that selective HVEM–ligand interactions are important in resistance to mucosal bacterial infection and in prevention of liver inflammation in a context where all members of the protein network were present and only selective interactions were disrupted.

## Results

### hHVEM–hLIGHT complex exists as a 3:3 assembly

**Figure 1.**
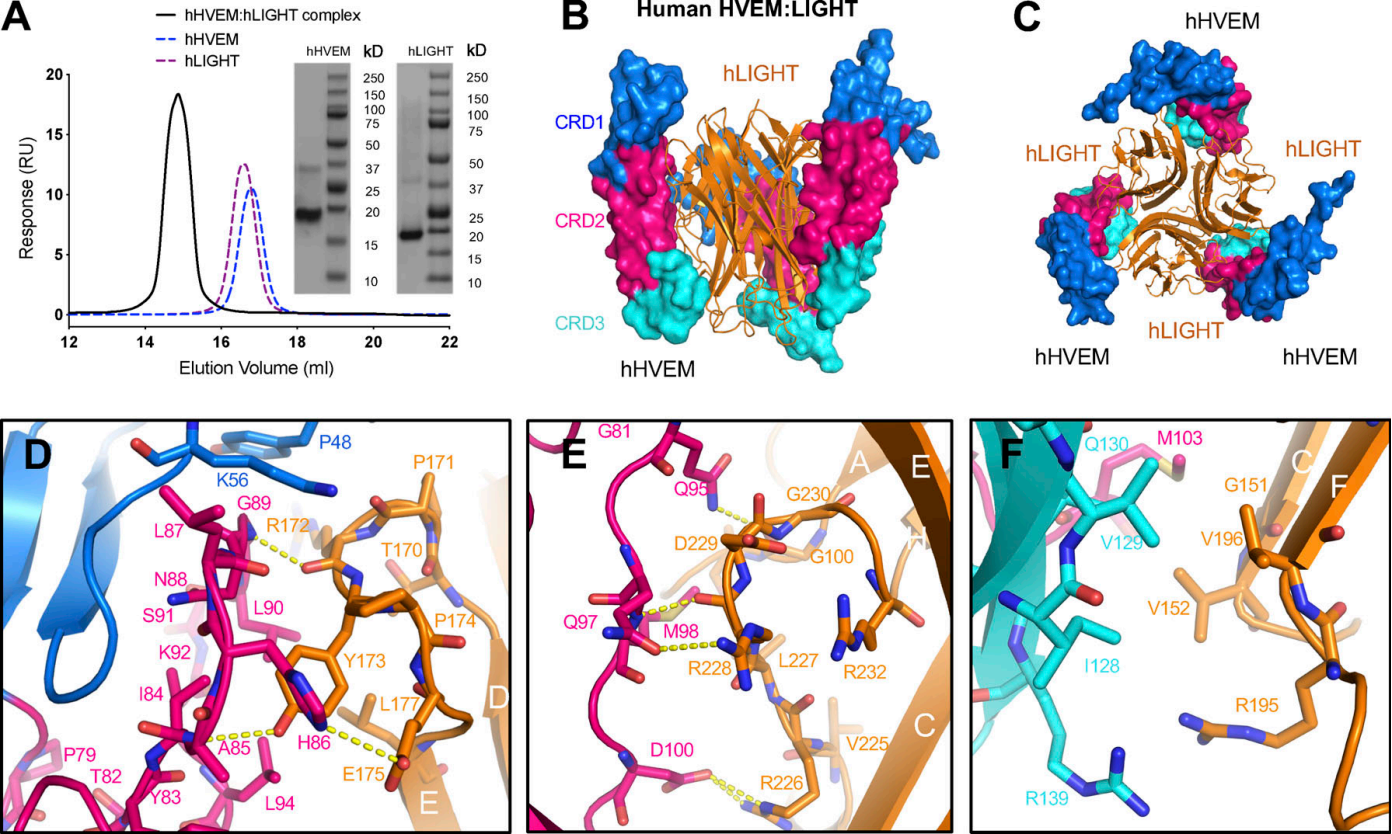
**Crystal structure of hHVEM–hLIGHT complex exhibits a 3:3 stoichiometry. (A)** The analytical SEC trace of hHVEM and hLIGHT mixtures reveals a significant peak of the complex corresponding to the molecular weight ∼100 kD. The SDS-PAGE results indicate hHVEM and hLIGHT were purified to near homogeneity. Note that in the SDS gel, LIGHT trimers dissociate. RU, response units. **(B and C)** The hHVEM is shown as a surface, and each CRD domain is colored separately as indicated in the figure. The trimeric hLIGHT protein is shown as an orange ribbon in the figure. The side view (B) and bottom view (C) of the hHVEM–hLIGHT complex are shown. **(D–F)** The detailed interaction interface between hHVEM and hLIGHT. The hHVEM CRD1, CRD2, and CRD3 residues are colored as marine, hot pink, and cyan, respectively. hLIGHT residues are colored as orange. The hydrogen bonds between hHVEM and hLIGHT are indicated as dashed lines.

The extracellular domains of human LIGHT (denoted as hLIGHT; ∼18 kD for the monomer and ∼54 kD for the homotrimer) and hHVEM (∼15 kD) were purified to homogeneous, monodisperse species as indicated by analytical size exclusion chromatography (SEC; Fig. 1 A). Mixing equal molar equivalents of hLIGHT and hHVEM monomers resulted in a single species with an apparent molecular weight of ∼100 kD, consistent with the formation of a 3:3 stoichiometric hHVEM–hLIGHT assembly in solution (Fig. 1, A–C).

**Figure S1.**
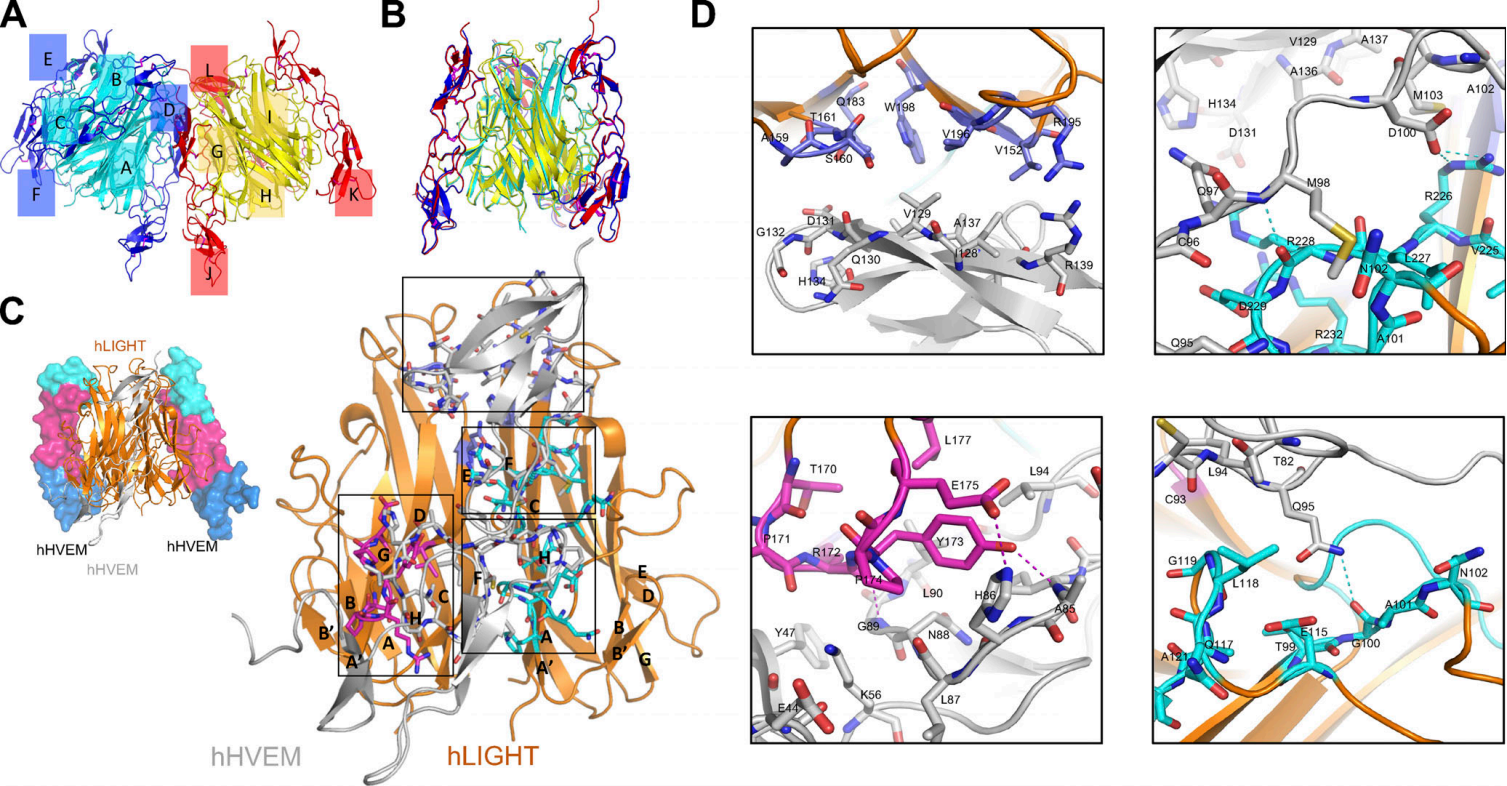
**Overall structure of the hHVEM–hLIGHT complex and the binding interface between hHVEM and hLIGHT. (A and B)** One asymmetry unit contains six independent chains of hLIGHT (cyan and yellow cartoon) and six independent chains of hHVEM (blue and red cartoon) forming two independent 3:3 hHVEM–hLIGHT complexes. Each chain is labeled in the figure. **(A)** Side view of the two hHVEM–hLIGHT complexes in one asymmetry unit. **(B)** Side view of the superimposition result of the two hHVEM–hLIGHT complexes. **(C)** The overall structure of the hHVEM–hLIGHT complex (top left) and magnified view of one copy hHVEM binding to two adjacent hLIGHT monomers (bottom right). hLIGHT is shown as an orange cartoon. hHVEM is presented as gray cartoon for one copy and a CRD colored surface for two copies. **(D)** Magnified views of the binding interface between hHVEM and hLIGHT. The residues from the “upper” region of the hHVEM–hLIGHT complex are shown as marine color sticks on the top left panel. The residues from the AA″ and GH loops part of the “lower” region of the hHVEM–hLIGHT interface are shown as cyan sticks on the top right and bottom right panels. The residues from the DE loop part of the lower region of the hHVEM–hLIGHT interface are shown as magenta sticks on bottom left panel. The residues of hHVEM contributing to the interface are presented as gray sticks. Shown are the interaction interface of the “upper” region between hLIGHT and hHVEM (top left panel), the interaction interface between the GH loop of hLIGHT and hHVEM (top right panel), the interaction interface between the DE loop of hLIGHT and hHVEM (bottom left panel), and the interaction interface between the AA′ loop of hLIGHT and hHVEM (bottom right panel).

The crystal structure of the hHVEM–hLIGHT complex was determined to the resolution of 2.30 Å by molecular replacement using Protein Data Bank (PDB) entries 4KG8 (hLIGHT) and 4FHQ (hHVEM) as starting search models (Table 1). The asymmetric unit of the hHVEM–hLIGHT crystals contains six independent chains of hLIGHT and six independent chains of hHVEM, which form two classical 3:3 TNF–TNFR hexameric assemblies with threefold symmetry (Fig. S1, A–C); a single 3:3 TNF–TNFR hexameric assembly is consistent with SEC analysis. The hHVEM ectodomain is composed of four cysteine-rich domains (CRDs), while hLIGHT forms a compact homotrimeric structure. In the hexameric assembly, CRD1, CRD2, and CRD3 of hHVEM engage hLIGHT via surfaces contributed by two adjacent hLIGHT protomers (Figs. 1 B and S1 C). The two independent hHVEM–hLIGHT hexameric complexes exhibit similar overall structures with a root mean square deviation of 1.8 Å for 742 aligned C_α_ atoms. The regions with the greatest structural divergence reside in the N and C termini of the proteins, which do not directly contribute to the binding interface. The hHVEM–hLIGHT recognition interfaces are highly similar within and between the two complexes (Fig. S1 B), and the following discussion is based on the hLIGHT G and H chains and hHVEM J chain (Fig. S1 A).

**Table 1.** Data collection and refinement statistics

	hHVEM–hLIGHT	hHVEM–hLIGHT–hCD160	mHVEM
**Data collection**			
Wavelength used (Å)	1.075	0.97931	0.97931
Resolution range (Å)	2.30-50.00 (2.30-2.34)	3.50-50.00 (3.50-3.83)	2.10-50.00 (2.10-2.14)
Space group	P2_1_2_1_2_1_	I23	P4_1_2_1_2
Unit cell (Å)	a = 111.7, b = 113.6, c = 163.3	a = b = c = 214.7	a = b = 64.7, c = 69.0
Unique reflections (N)	92,792	20,868	8,989
Redundancy	10.8(10.7)	20.7 (17.9)	13.5 (9.9)
Completeness	99.9(99.7)	99.9 (100)	99.5 (99.1)
I/sigma	22.7 (3.1)	16.1 (2.2)	17.1 (2.2)
R_merge_^a^	0.125 (0.936)	0.191 (1.674)	0.135 (0.938)
CC_1/2_	N/A	0.999 (0.676)	0.999 (0.943)
**Refinement**			
Resolution range (Å)	2.30-48.92 (2.30-2.36)	3.50-19.93 (3.50-3.59)	2.10-20.00 (2.10-2.16)
R_work_^b^	0.188 (0.245)	0.257 (0.370)	0.212 (0.355)
R_free_	0.231 (0.270)	0.285 (0.293)	0.257 (0.328)
Average B factor (Å^2^)	38.4	139.9	55.5
Rms bond (Å)	0.021	0.005	0.018
Rms angles (°)	2.081	1.290	1.928
PDB code	4RSU	7MSG	7MSJ

Parentheses indicate statistics for the highest-resolution bin. Rms, root mean square. N, numbers.

a R_merge_ = Σ_hkl_Σ_i_|I_i_(hkl) − <I(hkl)>|/Σ_hkl_Σ_i_I_i_(hkl).

b R_work_ = Σ|F_c_ − F_o_|/ΣF_o_.

### The binding interface between hHVEM and hLIGHT

The structure of the hHVEM–hLIGHT complex shows that HVEM CRD1 and CRD2 domains interact with the DE, AA′ and GH loops of LIGHT, while HVEM CRD3 interacts with LIGHT CD and EF loops (Fig. 1, D–F; and Fig. S1, C and D).

**Figure S2.**
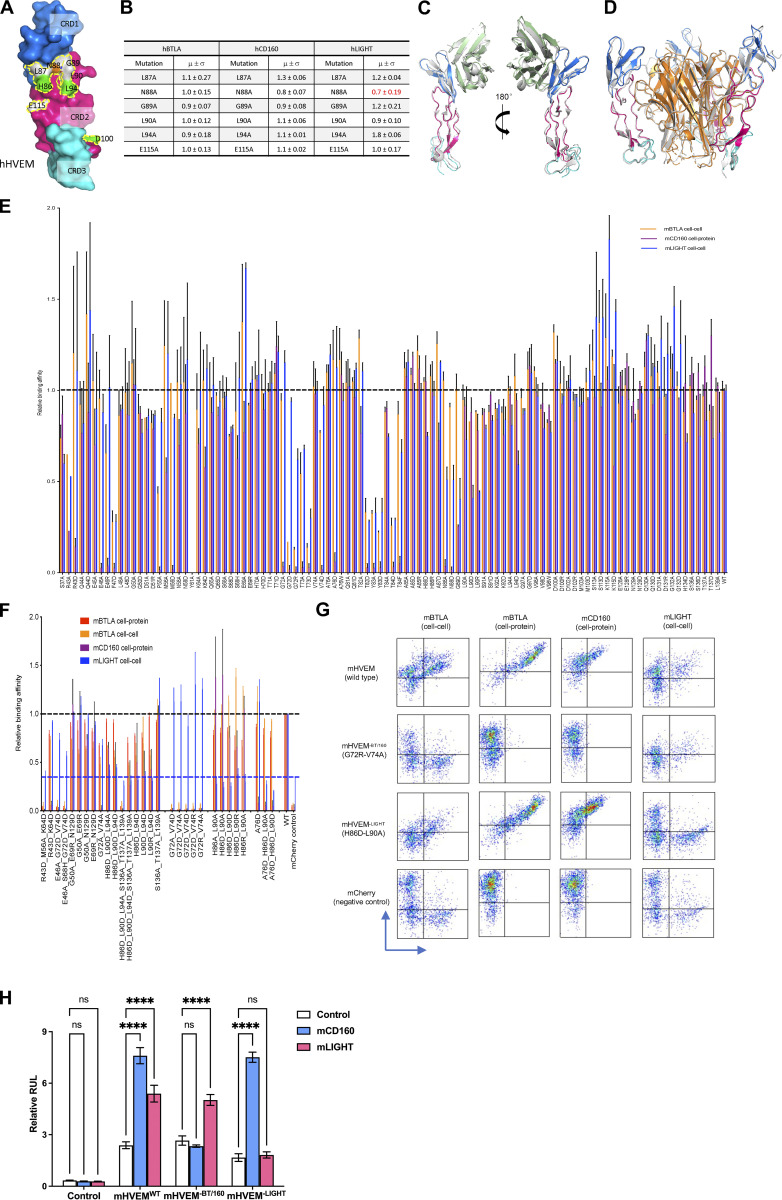
**Relative binding affinities of HVEM mutants with BTLA, CD160, and LIGHT. (A and B)** The hHVEM mutants were expressed on the cell surface and stained by hCD160, hBTLA, and hLIGHT proteins. The relative binding affinities were measured by flow cytometry. Error bars represent results from at least triplicates. **(A)** Positions of the hHVEM mutation residues. The residue hLIGHT Y173 (highlighted in yellow) is shown as yellow stick in the structure. **(B)** Relative binding affinities of the hHVEM mutants. **(C)** Superimposition of hHVEM–hCD160 from the ternary complex with the hHVEM–hCD160 complex alone (gray cartoon, PDB entry 6NG3). **(D)** Superimposition of hHVEM–hLIGHT from the ternary complex with the hHVEM–hLIGHT complex alone (gray cartoon, PDB entry 4RSU). **(E)** Relative binding affinities of mHVEM single-residue muteins with its ligands. Error bars represent results from at least triplicates and are shown as black sticks. The gray dashed line marks the averaged normalized affinities of WT mHVEM with mBTLA, mCD160, and mLIGHT. **(F)** Relative binding affinities of mHVEM multiple-residue muteins with its ligands. Error bars represent results from at least triplicates and are shown as thinner sticks with corresponding colors. The gray dashed line marks the averaged normalized affinities of WT mHVEM with mBTLA, mCD160, and mLIGHT. Blue dashed line marks the average background noise resulting from the non-specific interaction of negative control mCherry with mLIGHT.**(G)** Representative flow cytometry results. The vertical axis is the mCherry fluorescence indicating mHVEM-expressing cells, and the horizontal axis is the green fluorescence staining of fusion proteins or binding partner–expressing cells, as indicated. **(H)** Ligand-selective mHVEM mutein signaling. 293T cells were cotransfected with mHVEM^WT^, mHVEM^−BT/160^, mHVEM^−LIGHT^, or control (vector only) along with an NF-κB–driven luciferase (NF-κB-Luc) vector. mHVEM/NF-κB-Luc–expressed cells were co-cultured with control or mCD160- or mLIGHT-transfected 293T cells as indicated. Luciferase activity was measured after 18 h. RLU (relative light units) is the ratio of Firefly luciferase luminescence to *Renilla* luciferase luminescence. Data shown are mean ± SD. ****, P < 0.0001 for two-way ANOVA. Data are representative of two independent experiments.

The interaction between the hHVEM CRD2 and hLIGHT DE loop appears to be important for hHVEM–hLIGHT recognition, as it contributes multiple potential polar contacts. The main-chain amide group of hHVEM A85 (position numbered with initiation codon = 1) forms a hydrogen bond with the side-chain hydroxyl group of hLIGHT Y173 (Figs. 1 D and S1 D), consistent with the behavior of the Y173F mutation in hLIGHT, which significantly diminishes the binding of hLIGHT with hHVEM (Rooney et al., 2000). hHVEM N88 does not directly contact hLIGHT Y173 but is relatively close, and the hHVEM N88A mutation attenuated binding to hLIGHT (Fig. S2, A–D). Notably, the residues analogous to LIGHT Y173 in FasL, TL1A, TRAIL, TNFα, and LTα are conserved, and these tyrosines are also important for DE loop–mediated receptor binding, whereas the homologous residues in receptor activator of NF-κB ligand, OX40L, CD40L, and 4-1BBL are not tyrosines and are not critical for receptor binding, indicating diverse mechanisms of binding among different TNF ligands and receptors. The hHVEM G89 main-chain amide group forms a hydrogen bond with the main-chain oxygen of hLIGHT R172 (Figs. 1 D and S1 D). HVEM H86 side-chain imidazole functionality makes a polar contact with the side-chain carboxyl group of hLIGHT E175 (Figs. 1 D and S1 D). It was previously reported that the hHVEM H86I mutation dramatically reduced binding to hLIGHT (Shrestha et al., 2020).

hHVEM CRD2 forms four additional polar contacts with the GH loop of hLIGHT (Figs. 1 E and S1 D). The hHVEM Q97 side-chain oxygen forms a polar contact with the hLIGHT R228 side-chain. The hHVEM M98 backbone amide group contacts the backbone oxygen of hLIGHT R228, and the side-chain carboxyl group of hHVEM D100 forms two polar contacts with the side-chain guanidinium group of hLIGHT R226 (Figs. 1 E and S1 D). The hHVEM D100R mutation resulted in undetectable binding with hLIGHT (Shrestha et al., 2020). The AA′ loop from the lower region of CRD2 contributes only a single polar contact, formed by the main-chain oxygen from G100 of hLIGHT and the side-chain amide group of hHVEM Q95 (Figs. 1 E and S1 D).

hHVEM CRD3 residues, including I128-G132, H134, and A136-R139, participate in interactions with G151-V152 and A159-T161 from the CD loop, as well as residues Q183, R195-V196, and W198 from the EF loop of hLIGHT (Fig. S1, C and D). Examination of the structure in this region reveals no polar contacts between hHVEM and hLIGHT. A modest hydrophobic interface is formed by the packing of the side chains of hHVEM residues I128 and V129 against the side chains of hLIGHT V152 and V196 (Figs. 1 F and S1 D).

### Structure of the hHVEM–hLIGHT–hCD160 ternary complex

**Figure 2.**
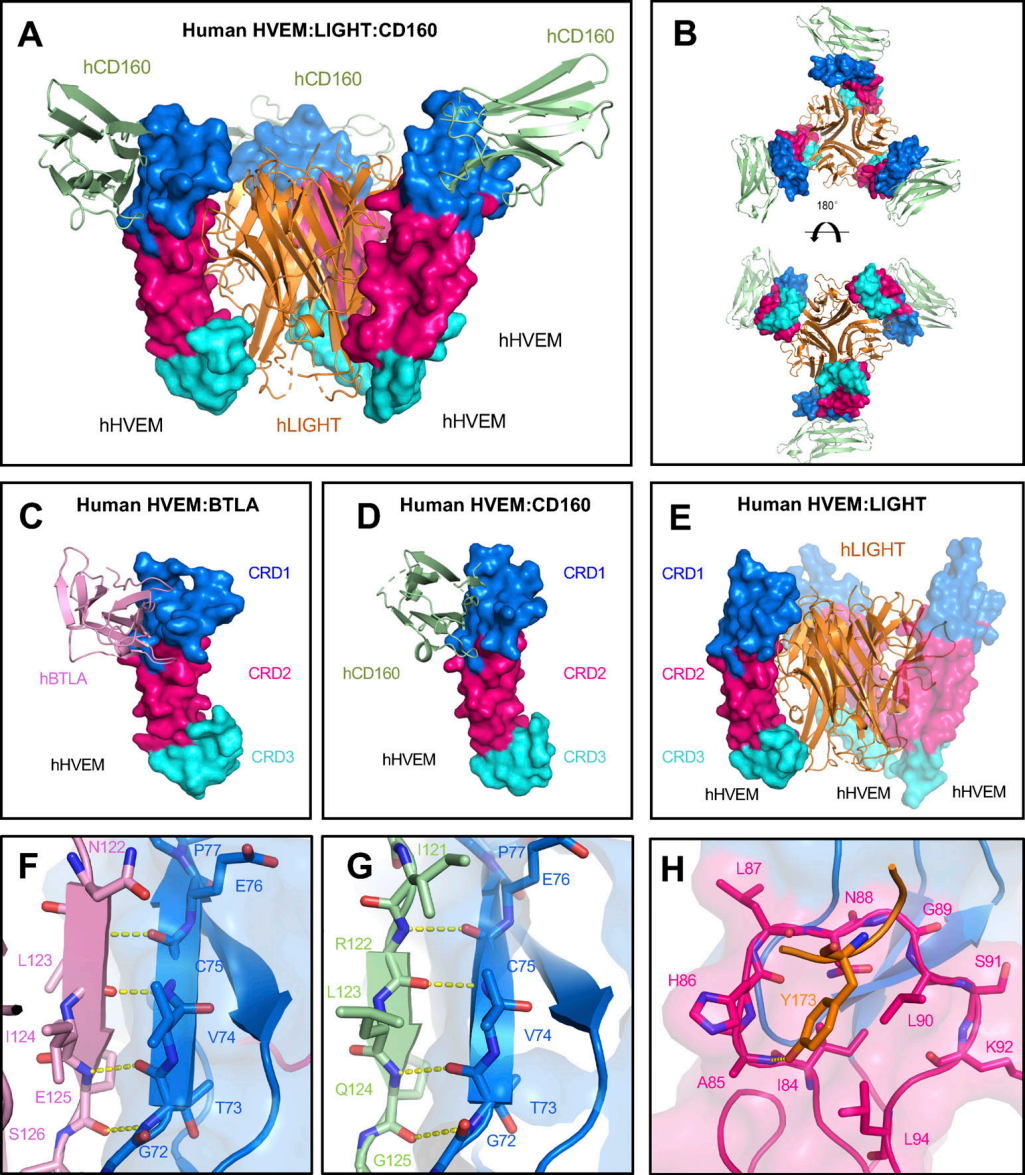
**Overall structure of hHVEM–hLIGHT–hCD160 ternary complex and critical interaction interfaces of HVEM binding to BTLA, CD160, and LIGHT.** **(A and B)** Structure of the hHVEM–hLIGHT–hCD160 ternary complex indicates hCD160 and hLIGHT can interact simultaneously with hHVEM. The side view (A) and the top/bottom views (B) of the ternary complex are shown. **(C)** Structure of hHVEM–hBTLA (PDB entry 2AW2). **(D)** Structure of hHVEM–hCD160 (PDB entry 6NG3). **(E)** Structure of hHVEM–hLIGHT (PDB entry 4RSU). These structures indicate hBTLA and hCD160 bind to similar surfaces on hHVEM, whereas hLIGHT binds to a different surface on hHVEM. **(F–H)** Detail binding interfaces between hHVEM and its binding ligands hBTLA, hCD160, and hLIGHT, respectively. The hHVEM CRD1 and CRD2 domains are colored as marine and hot pink, respectively.

It was previously shown that LIGHT and the IgSF ligands do not compete for binding to HVEM (Cai et al., 2008; Liu et al., 2019), suggesting the potential for forming a ternary complex. Therefore, we set out to solve the crystal structure of hHVEM–hLIGHT–hCD160 (human CD160 is denoted as hCD160) complex (PDB entry 7MSG). Accordingly, we determined the structure of this complex to 3.5 Å resolution by molecular replacement using CD160 (PDB entry 6NG9) and the hHVEM–hLIGHT complex described above (PDB entry 4RSU) as search models (Fig. 2, A and B). The asymmetric unit contains three copies of each hHVEM, hLIGHT, and hCD160, forming a ternary complex with 3:3:3 stoichiometry. Within the ternary assembly, hHVEM and hLIGHT exhibit the classical 3:3 TNF–TNFR assembly, with contacts that are very similar to the structure of the hHVEM–hLIGHT binary complex described above. The hHVEM–hLIGHT complex forms the core of the ternary complex, with each hHVEM CRD1 further binding a single molecule of hCD160 in a manner similar to that observed in the structure of the hHVEM–hCD160 binary complex (Fig. 2, A and D and Fig. S2 C). Notably, the structures of hHVEM–hLIGHT–hCD160 and hHVEM–hCD160 complexes relied on the use of a single chain hCD160–hHVEM fusion protein, as the relatively weak interaction of hCD160–hHVEM (7.1 ± 0.9 µM) does not support the stable complex formation in solution (Liu et al., 2019). The crystal structure of the hHVEM–hLIGHT–hCD160 complex provides direct evidence that hLIGHT and hCD160 can simultaneously engage hHVEM, resulting in a higher-order assembly with the potential of coordinated signaling through both hHVEM and hCD160. Notably, the simultaneous interaction of hCD160 and hLIGHT with hHVEM alters the local organization of hCD160, as engagement of hHVEM with trimeric hLIGHT may enforce close proximity of up to three hCD160 molecules with distinct geometric organization, as compared with the engagement of hCD160 and hHVEM in the absence of hLIGHT.

Crystal structures and complementary mutagenesis studies of hHVEM–hCD160 and hHVEM–hBTLA (human BTLA is denoted as hBTLA) complexes demonstrated that both hCD160 and hBTLA mainly bind to CRD1 on hHVEM (Fig. 2, C and D; Compaan et al., 2005; Liu et al., 2019). In contrast, the crystal structure of the hHVEM–hLIGHT complex shows hLIGHT binds to CRD2, CRD3, and a small part of CRD1 on hHVEM (Fig. 2 E). Crystal structures of hHVEM in complex with hBTLA and hCD160 highlight an anti-parallel intermolecular β-strand interaction, in which the β-strand composed of residues G72-P77 from CRD1 in hHVEM contacts the edge β-strands in hBTLA and hCD160 through canonical main-chain-to-main-chain β-sheet hydrogen bonds (Fig. 2, F and G). This pattern of hCD160 interactions with hHVEM is conserved in the ternary hHVEM–hLIGHT–hCD160 complex. Mutations of residues within this intermolecular β-strand (G72-P77) in HVEM CRD1 significantly altered the binding affinities (Shrestha et al., 2020), while hHVEM CRD2 mutations do not significantly alter the affinities to hCD160 and hBTLA. In contrast, HVEM CRD2 mutations, particularly the HVEM residues forming the concave cavity surrounding hLIGHT Y173, significantly affect hHVEM–hLIGHT binding (Fig. 2 H). Because both hCD160 and hBTLA bind to similar epitopes on hHVEM CRD1 (Compaan et al., 2005; Liu et al., 2019), it is likely that hHVEM, hLIGHT, and hBTLA are able to form a ternary complex similar to the trimolecular complex of hHVEM–hLIGHT–hCD160 we have determined.

### Structure-guided mutagenesis of mHVEM mutants

**Figure 3.**
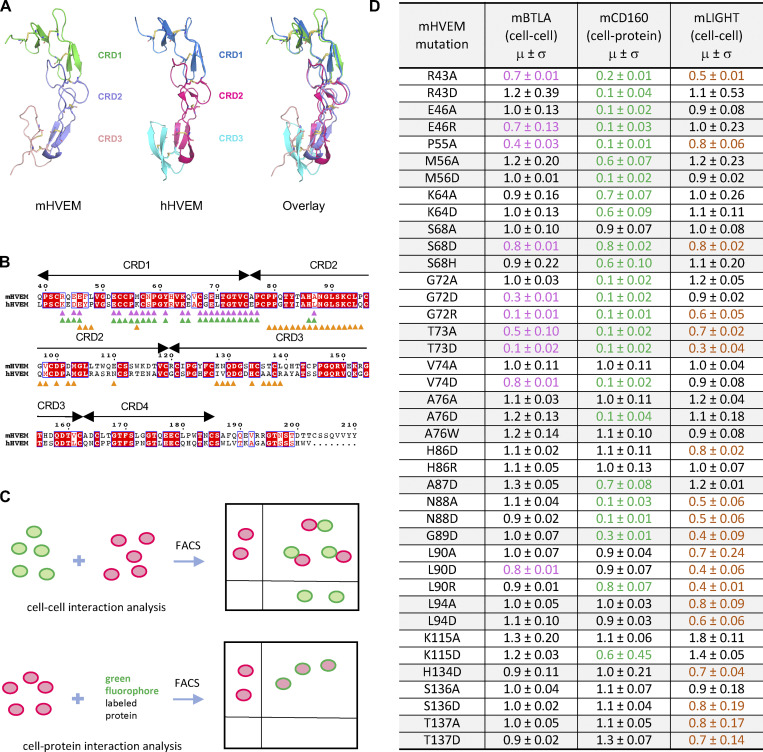
**Structure and mutagenesis screen of mHVEM. (A)** Structures of mHVEM and hHVEM and their comparison. The disulfide bonds of HVEM are shown as sticks and each HVEM CRD is colored differently. **(B)** Sequence alignment of mHVEM and hHVEM. The homologous residues are highlighted in red. The residues of hHVEM directly involved in the interface with hBTLA, hCD160, and hLIGHT are marked by magenta, green, and orange triangles, respectively. **(C)** The schematic figure shows two ways to determine the relative binding affinities of mHVEM mutants. The cell–cell method measures the percentages of double-positive cells in the mixtures. The cell–protein method measures the percentages of green-fluorophore–stained mHVEM-mCherry–expressing cells. **(D)** Relative binding affinities of mHVEM mutants with its ligands are shown in the table. Both mBTLA and mLIGHT binding to mHVEM was assessed by the cell–cell method. The mCD160 binding to mHVEM was tested by cell–protein method. Error bars represent results from at least triplicates. All mHVEM mutants with ≥20% binding reduction to a particular query are colored differently to indicate their reduced affinities.

The mHVEM (PDB entry 7MSJ) structure was determined to 2.10 Å resolution by molecular replacement using hHVEM (PDB entry 4FHQ) as the search model. mHVEM and hHVEM structures are similar, with a root mean square deviation of 2.7 Å for 97 aligned C_α_ atoms, with the biggest differences in CRD3 (Fig. 3, A and B). Based on structural and sequence alignments between hHVEM and mHVEM, the solvent-accessible mHVEM residues close to the putative binding interfaces were mutated to dissect the interaction network and enable in vivo HVEM functional studies.

The relative binding affinities of mHVEM mutants with mBTLA and mLIGHT (mouse BTLA and mouse LIGHT are denoted as mBTLA and mLIGHT, respectively) were evaluated by a cell–cell interaction assay (Fig. 3 C). The relative binding affinities of mHVEM mutants for mCD160 (mouse CD160 is denoted as mCD160) binding were screened using a cell-soluble protein assay because of low surface expression of the CD160 protein. A total of 52 mHVEM surface residues within or close to the likely ligand-binding interfaces were individually mutated to different amino acids to probe the effect on ligand binding and to identify variants with selective ligand recognition (Fig. 3 D). For example, alteration of mHVEM G72 or V74 to aspartic acid attenuated binding to both mBTLA and mCD160, but not binding to mLIGHT; the mHVEM R43D, M56D, or A76D mutations decreased binding to mCD160, but not mBTLA and mLIGHT; and the mHVEM H86D, L90A, L94A, and L94D mutations compromised the interaction with mLIGHT, but not mBTLA or mCD160 (Figs. 3 D and S2 E).

**Figure 4.**
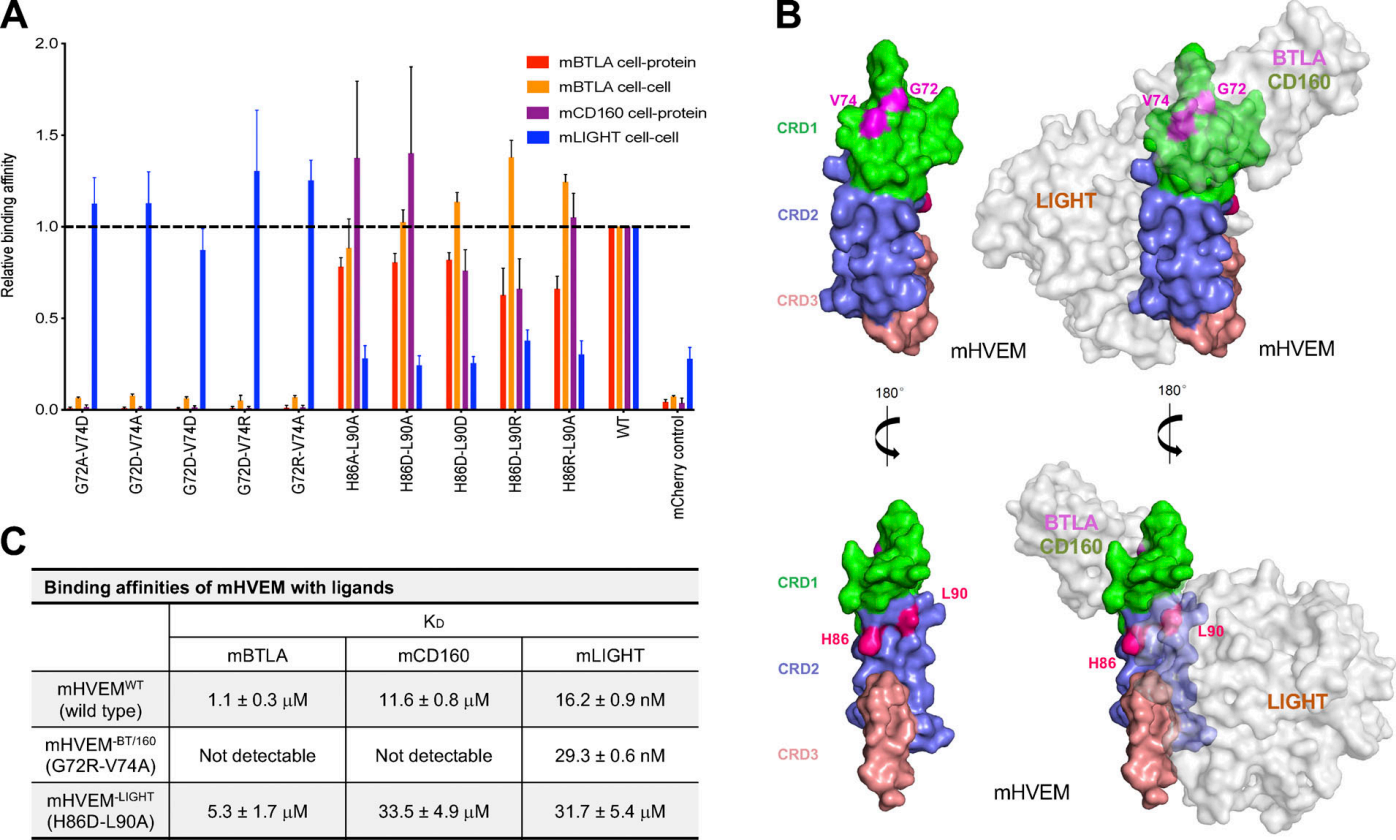
**The engineered mHVEM mutants have binding selectivity. (A)** The relative binding affinities of mHVEM mutants with mBTLA, mCD160, and mLIGHT as measured by cell–cell or cell–protein methods. Error bars represent results from at least triplicates. The gray dashed line marks the averaged normalized affinities of WT mHVEM with mBTLA, mCD160, and mLIGHT. Blue dashed line marks the average background noise resulting from the non-specific interaction of negative control mCherry with mLIGHT. **(B)** The locations of the mutated residues on mHVEM. mHVEM is shown as a surface, with each CRD colored differently; G72, V74, H86, and L90 are marked on the mHVEM surface. Ligands BTLA, CD160, and LIGHT are modeled based on the HVEM structures and are shown as labeled gray surfaces. **(C)** The binding affinities of mHVEM^WT^ (WT mHVEM), mHVEM^−BT/160^ (mHVEM G72R-V74A double mutein), and mHVEM^−LIGHT^ (mHVEM H86D-L90A double mutein) with mBTLA, mCD160, and mLIGHT as measured by Octet biolayer interferometry technology.

To further modulate the selectivity toward mLIGHT or mBTLA/mCD160, mHVEM mutations with similar binding properties were combined (Figs. 4 A and S2 F). For example, the combination of the G72 and V74 mutations completely eliminated binding to both mBTLA and mCD160 but did not appreciably impact mLIGHT binding in the flow cytometry–based binding assays. Various pairwise combinations of mutations of H86, L90, and L94 eliminated mLIGHT binding but did not substantially impact binding to mBTLA or mCD160 (Fig. 4 A; and Fig. S2, F and G). Thus, these compound mutations resulted in several additional mHVEM variants with considerable binding selectivity. Although triple mutation of H86, L90, and L94 removed mLIGHT binding, it also dramatically reduced binding to mBTLA and mCD160 (Fig. S2 F). Not surprisingly, other combinations of mutations also reduced the binding to all ligands, such as the mHVEM R43D-M56A-K64D triple mutation (Fig. S2 F).

Residues G72 and V74 contribute to the binding interface of the hHVEM–hCD160 and hHVEM–hBTLA complexes (Fig. 2, F and G; and Fig. 4 B), whereas H86 and L90 resides are within the hHVEM–hLIGHT interface in close proximity to hLIGHT Y173, based on the hHVEM–hLIGHT structure (Figs. 2 H and 4 B). The mHVEM G72R-V74A double mutation exhibited no binding to mBTLA or mCD160, while it retained WT binding to mLIGHT in our cell–cell and cell–protein interaction system (Fig. 4 A; and Fig. S2, F and G). This mHVEM mutant was selected for further analysis and is designated as mHVEM^−BT/160^, denoting loss of BTLA and CD160 binding. The mHVEM H86D-L90A double mutation showed no binding to mLIGHT and WT binding to mBTLA and mCD160 (Fig. 4 A; and Fig. S2, F and G). This mHVEM H86D-L90A mutant is thus designated as mHVEM^−LIGHT^, denoting loss of LIGHT binding. Both mHVEM^−BT/160^ and mHVEM^−LIGHT^ proteins were expressed in soluble form, and their ligand binding was measured by surface plasmon resonance. The mHVEM^−BT/160^ eliminated binding to both mBTLA and mCD160, while it still retained close to WT binding to mLIGHT (Fig. 4 C). The mHVEM^−LIGHT^ had approximately fivefold and threefold reduced binding to mBTLA and mCD160, respectively, but had more than a three-log-fold decrease in binding to mLIGHT (Fig. 4 C). We also determined if signaling in vitro by mHVEM muteins was ligand selective. WT mHVEM or mHVEM muteins were cotransfected in 293T cells with an NF-κB–driven luciferase vector. Transfectants of 293T cells with either mCD160 or mLIGHT activated downstream NF-κB signaling of WT mHVEM (Fig. S2 H). Activation was selective, however, as mHVEM^−BT/160^ transfectants could be signaled by LIGHT, but not by CD160-expressing cells, and the opposite was true for cells expressing mHVEM^−LIGHT^.

### mHVEM^−LIGHT^ mice are more susceptible to *Yersinia *infection

**Figure S3.**
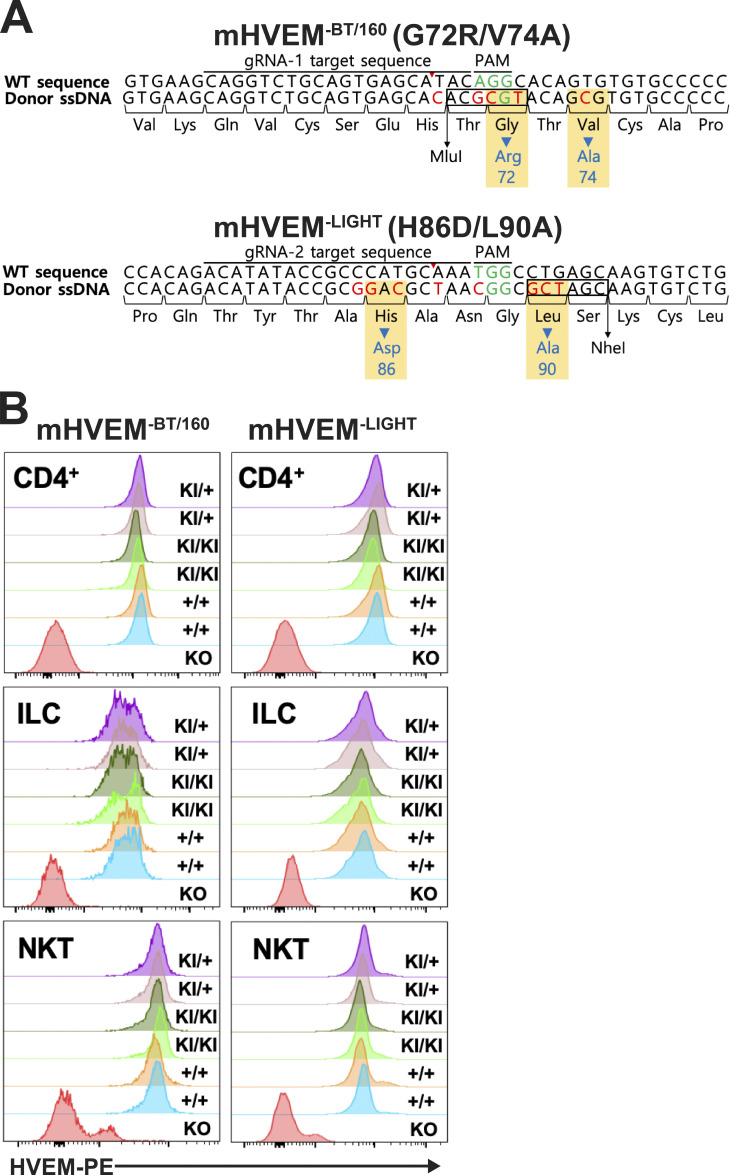
**Normal surface HVEM expression in mHVEM mutant mice. (A)** Schematic of nucleotide sequences of the HVEM gene in mHVEM mutant mouse strains (G72R/V74A: mHVEM^−BT/160^, loss of BTLA and CD160 binding; H86D/L90A: mHVEM^−LIGHT^, loss of LIGHT binding) that were generated by CRISPR-Cas9 editing of exon 3 of the *Tnfrsf14* locus. Red letters indicate mutated nucleotides. Green letters indicate protospacer adjacent motif (PAM) sequence. Blue letters indicate mutated amino acids. Black box shows restriction enzyme sites. **(B)** HVEM surface expression level of splenic CD4^+^ T cells, ILCs (CD3^−^Lin^−^CD90.2^+^), and iNKT cells (TCRβ^+^, CD1d tetramer^+^) from the indicated mice were determined by flow cytometry. HVEM-knockout mouse is a negative control for HVEM staining. KI, KI allele. ssDNA, single-stranded DNA.

We tested the role of the mHVEM muteins mHVEM^−BT/160^ (G72R-V74A) and mHVEM^−LIGHT^ (H86D-L90A) in vivo. We used the CRISPR-Cas9 system to generate two knockin (KI) mouse strains (Fig. S3 A). KI homozygous mice having either HVEM mutein were born at the expected frequency with normal size and maturation. Immune cells from homozygous KI mice from either strain expressed a normal surface level of HVEM in different cell types, including splenic CD4^+^ T cells, invariant natural killer T (iNKT) cells, and ILCs (Fig. S3 B).

**Figure 5.**
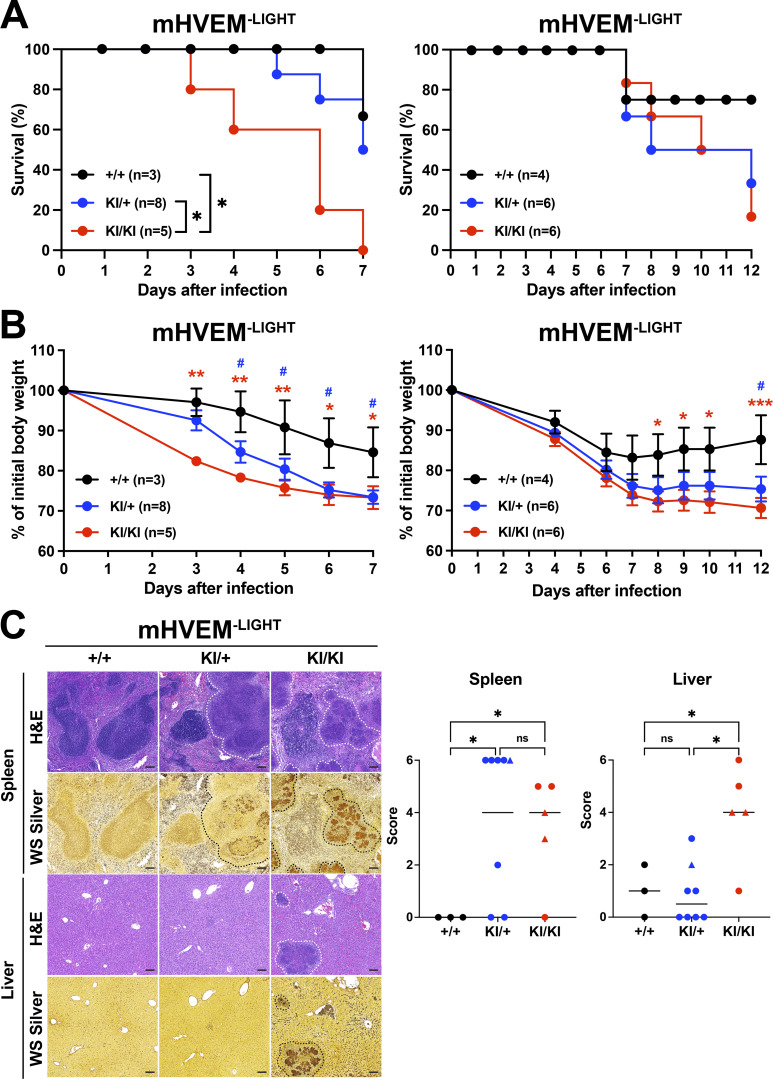
**mHVEM^−LIGHT^ mice are more susceptible to *Y. enterocolitica* infection.** Co-housed littermates were infected with 1.0 × 10^8^ *Y. enterocolitica*. **(A)** Survival curves in day 7 and day 12 experiments. *, P < 0.05 for log-rank test. **(B)** Changes in body weight (percentage of baseline). The weights of mice that died during the experiment were carried forward. Data shown are mean ± SEM. *, P < 0.05; **, P < 0.01; ***, P < 0.001 (+/+ vs. KI/KI) or ^#^, P < 0.05 (+/+ vs. KI/+) for two-way repeated measures ANOVA with Bonferroni’s multiple hypothesis correction. Because of the number of mice that could be handled, experimental data were done at different times with different bacterial cultures. **(C)** Representative H&E staining to detect necrotic areas and WS silver staining to detect bacteria in splenic and hepatic sections from the indicated mice at 7 d after infection. Scale bars, 100 µm. White dotted lines indicate necrotic areas, and black dotted lines indicate *Y. enterocolitica*. The histopathologic scores of H&E sections were evaluated as described in Materials and methods for all mice analyzed from day 7 experiment in A and B (left panels). Mice that survived to at least day 6 are indicated with circles, and mice that died earlier are indicated with triangles. Data shown are median. *, P < 0.05 for two-way ANOVA.

**Figure S4.**
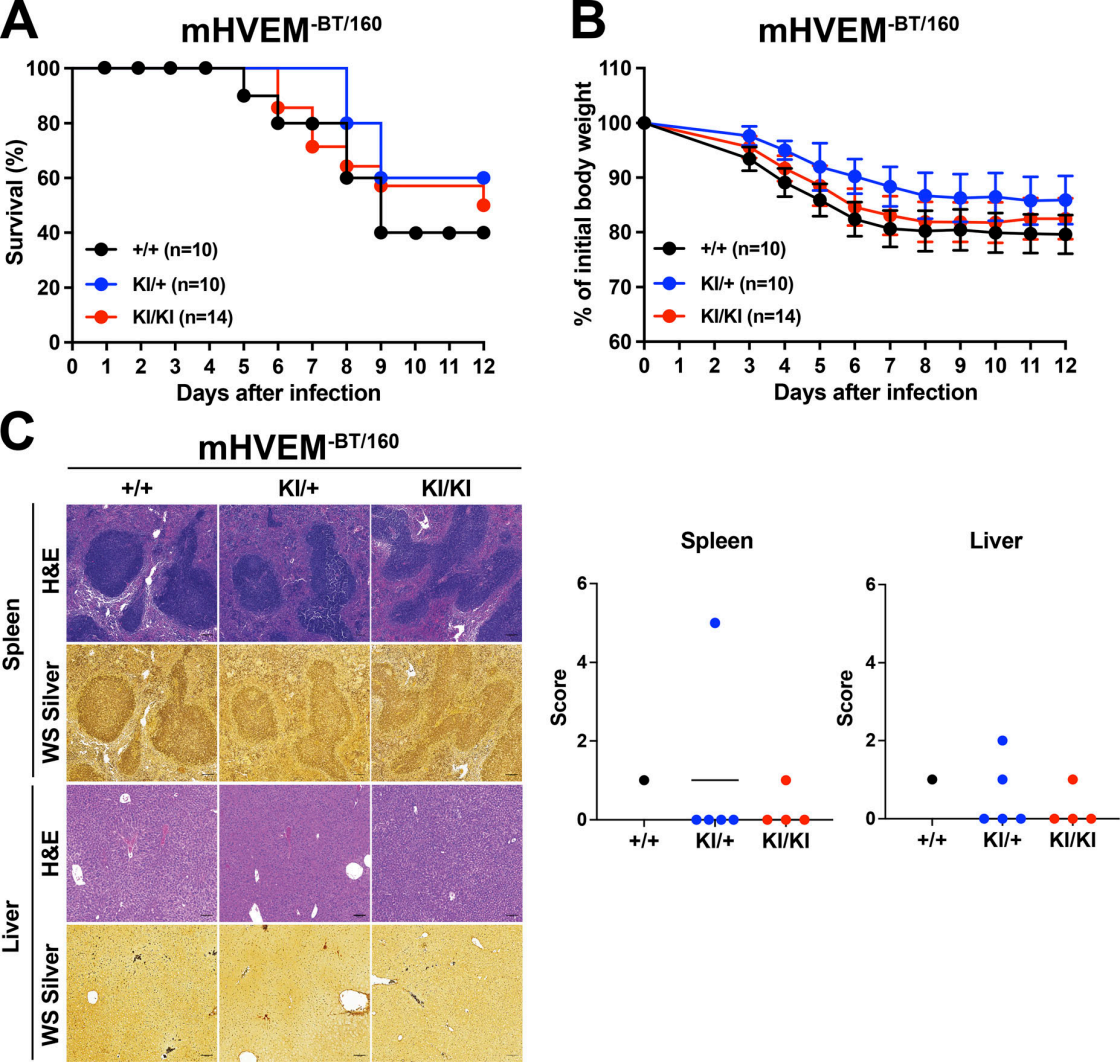
**mHVEM^-BT/160^ mice have a similar phenotype to controls during *Y. enterocolitica* infection.** Co-housed littermates were infected with 1.0 × 10^8^ *Y. enterocolitica*. KI, gene KI. **(A)** Survival curves in day 12 experiments. Data shown were analyzed by log-rank test. **(B)** Changes in body weight (percentage of baseline). The weights of mice that died during the experiment were carried forward. Data shown are mean ± SEM and analyzed by two-way ANOVA with Bonferroni’s multiple hypothesis correction. **(A and B)** Data represent pooled results from three independent experiments (*n* = 10–14 mice per group). **(C)** Representative H&E staining to detect necrotic areas and WS silver staining to detect bacteria in splenic and hepatic sections from the indicated mice at 7 d after infection. Scale bars, 100 µm. The histopathologic scores of H&E sections were evaluated as described in Materials and methods. Data shown are median for two-way ANOVA.

Previously, using conditional HVEM knockouts, we reported that HVEM signals in ILC3s are critical for host defense against oral infection with *Yersinia enterocolitica* (Seo et al., 2018). Importantly, the evidence from whole-body LIGHT-deficient mice suggested that this HVEM-mediated protection was dependent on LIGHT, not on BTLA or CD160. These data did not exclude a contribution by other aspects of this network. For example, LTβR-deficient mice were not tested, and LIGHT–LTβR interactions are also eliminated when the gene encoding LIGHT is deleted. To test the in vivo function of the HVEM muteins, mHVEM^−BT/160^ and mHVEM^−LIGHT^ mice were orally infected with *Y. enterocolitica*. The outcome of this infection can vary, and therefore, mice were followed either for 7 or 12 d in different experiments, depending on the severity of infection. Homozygous mHVEM^−LIGHT^ (KI/KI) mice displayed lower survival, although there was only a trend in this direction in the day 12 experiment with a milder infection. Clearly, there was more pronounced weight loss, without evidence for recovery, in the homozygous mHVEM^−LIGHT^ mice. Additionally, there were large areas of necrosis in the liver and spleen (Fig. 5). This severe disease outcome is similar to that observed in *Tnfsf14 *knockout mice (Seo et al., 2018), indicating LIGHT–LTβR interactions do not contribute to resistance or cannot overcome the effect of loss of LIGHT binding to HVEM expressed by ILC3s. Interestingly, heterozygous mHVEM^−LIGHT^ (KI/+) mice had an intermediate phenotype, with weight loss similar to homozygous mHVEM^−LIGHT^ mice, but they showed better survival than mHVEM^−LIGHT^ mice in the day 7 experiment, as well as reduced necrotic areas and decreased bacterial foci in the liver, but not in the spleen. Considering that LIGHT binding induces a trimerization of HVEM that likely enhances signaling, an intermediate phenotype might be expected in KI/+ heterozygous mice that would form fewer WT HVEM trimers. In a separate group of *Y. enterocolitica* infections performed with mHVEM^−BT/160^ mice, animals homozygous for a gene encoding the HVEM mutein that does not bind either IgSF ligand responded similarly to WT mice (Fig. S4). There was increased weight loss and decreased survival in the WT mice in the series of experiments with mHVEM^−BT/160^ mice (Fig. S4) compared with WT controls in experiments with mHVEM^−LIGHT^ mice (Fig. 5). The key comparison, however, is between mHVEM mutein and WT mice within an experiment, and only mHVEM^−LIGHT^ showed a difference from the WT in the same experiment. Also, note that bacterial clearance was greatly diminished by histological analysis at day 7 in homozygous mHVEM^−LIGHT^ mice, which also had large necrotic areas, but not in homozygous mHVEM^−BT/160^ mice. Therefore, our data suggest that indeed LIGHT is the unique ligand for HVEM in protection from *Y. enterocolitica* and that LIGHT binding to the LTβR is not relevant in this context.

### mHVEM^−BT/160^ mice are more susceptible to hepatic injury

Previous studies have reported that *Btla^−/−^* or *Cd160^−/−^* mice are more susceptible to hepatic injury induced by concanavalin A or the synthetic glycosphingolipid α-galactosylceramide (αGalCer; Iwata et al., 2010; Kim et al., 2019; Miller et al., 2009). We focused on αGalCer because of its well-defined mechanism of action as a specific activator of iNKT cells, which are very abundant in intrahepatic lymphocyte populations. When mice are injected with αGalCer, iNKT cells are rapidly stimulated and produced many types of proinflammatory cytokines, including TNF, IFNγ, and IL-4, driving liver injury (Biburger and Tiegs, 2005; Wang et al., 2013). Furthermore, both BTLA and CD160 are expressed by iNKT cells, and both molecules served to attenuate production of inflammatory cytokines by iNKT cells during αGalCer-induced acute hepatitis (Kim et al., 2019; Miller et al., 2009), providing an example in which two HVEM-binding IgSF molecules are required in one cell type. The function of LIGHT in this model has not been reported.

**Figure 6.**
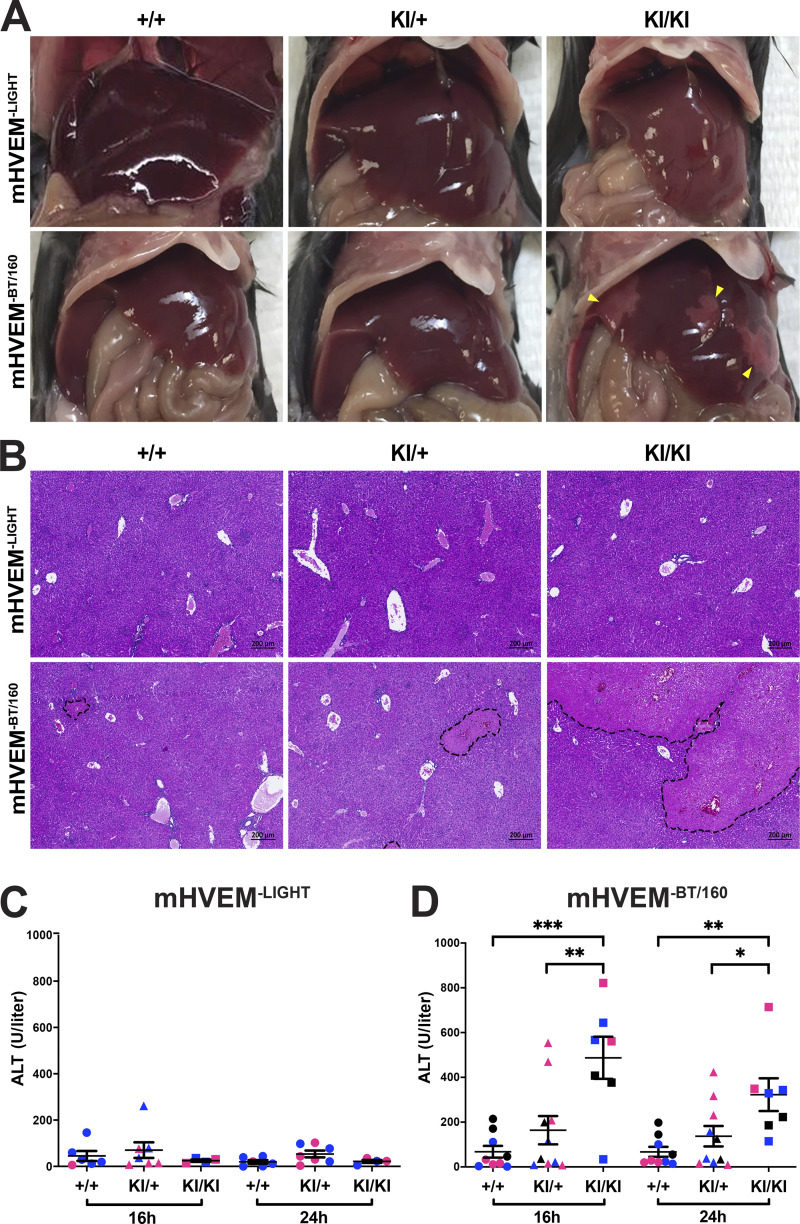
**Susceptibility to αGalCer-induced liver injury in mHVEM^−BT/160^ mice.** Co-housed littermates were injected with 2 µg αGalCer by the retro-orbital route. **(A)** Representative images of the liver 24 h after injection. Yellow arrowheads indicate necrotic areas. **(B)** Representative H&E staining of hepatic sections from the indicated mice 24 h after injection. Black dotted lines indicate the necrotic areas. Scale bars, 200 µm. **(C and D)** Serum ALT activity at 16 and 24 h from the indicated mice. Data shown are mean ± SEM. *, P < 0.05; **, P < 0.01; ***, P < 0.001 for one-way ANOVA. Data represent pooled results from at least two independent experiments; each experiment is labeled with different colored symbols (*n* = 4–10 mice per group; co-housed littermates). +/+ are indicated with circles; KI/+ are indicated with triangles; KI/KI are indicated with squares.

αGalCer was injected into female mHVEM^−LIGHT^ and mHVEM^−BT/160^ mice and controls. mHVEM^−LIGHT^ mice presented with a similar phenotype to controls, which at this dose induced only limited αGalCer-triggered liver damage and serum alanine aminotransferase (ALT) activity (Fig. 6, A–C). By contrast, larger white spots on the surface of liver and massive hepatic necrotic regions developed in mHVEM^−BT/160^ mice (Fig. 6, A and B). Consistently, serum ALT activity was elevated in mHVEM^−BT/160^ mice compared with littermate control or heterozygous (KI/+) mice (Fig. 6 D). Heterozygous mHVEM^−BT/160^ mice showed an intermediate phenotype, particularly with regard to the ALT measurement. Considering that the IgSF ligand–HVEM interaction is monomeric, this phenotype could reflect HVEM gene haploinsufficiency. These findings suggest that HVEM–BTLA and/or HVEM–CD160 engagement generated negative signaling in iNKT cells, thereby preventing severe αGalCer-induced liver injury and hepatitis.

## Discussion

HVEM and its ligands constitute an interacting network of cell surface proteins that affect many aspects of lymphocyte function, as well as the responses of numerous other cell types, including eosinophils, keratinocytes, epithelial cells, and macrophages, in the brain (Doherty et al., 2011; Herro et al., 2018; Shui et al., 2012; Zhu et al., 2016). To understand how HVEM functions in vivo in this network, and to develop therapeutics based on its mechanisms of action, one important tool is new mouse strains, including those that delete HVEM expression in certain cell types (Mintz et al., 2019; Seo et al., 2018), mutants that separate HVEM–ligand function from HVEM signaling, and expression of HVEM mutants with selective binding to only certain ligands. Here, we report the structures of human orthologues of members this network, including the ternary hHVEM–hLIGHT–hCD160 and binary hHVEM–hLIGHT complexes; we also report the structure of mHVEM in isolation. These structures guided mutagenesis studies that identified HVEM muteins with selective ligand binding. Additionally, we have tested these HVEM muteins in vivo in mouse strains. In this way, without eliminating expression of any member of the network, we provide data indicating that selective HVEM–ligand interactions are responsible for host defense from enteric bacterial infection and the prevention of liver inflammation.

**Figure S5.**
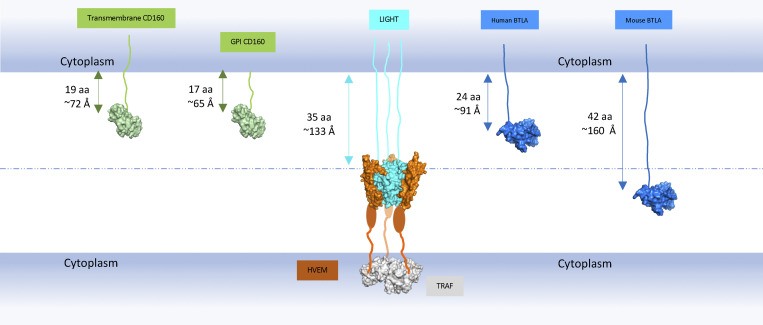
**Predicted maximum lengths of LIGHT, CD160, and BTLA stalk regions.** The globular domains of LIGHT, CD160, and BTLA are shown as surface structures and colored as cyan, green, and blue, respectively. The CRDs of HVEM are shown as orange surfaces, and the remainder of the CRD regions that were not visible in the structures are shown as orange ovals. The cytoplasmic TNF receptor associated-factor (TRAF) molecule is shown as a gray surface. The stalk regions that connect the extracellular globular domains to the transmembrane segments are shown as lines. The maximum lengths of the stalk regions are calculated as if they adopt the fully extended structures. The length of the GPI-anchored CD160 stalk region in the figure does not include the GPI length. This figure indicates that when human membrane LIGHT binds to HVEM, the longer stalk lengths of LIGHT may prevent BTLA and CD160 binding to HVEM.

In contrast to the homotrimeric structure of LIGHT, BTLA and CD160 proteins are monomers (Compaan et al., 2005; Zhu et al., 2016). Crystallographic and biochemical studies illustrated that hHVEM–hBTLA and hHVEM–hCD160 complexes are characterized by a 1:1 stoichiometry (Fig. 2, C and D; Compaan et al., 2005). Unlike trimeric LIGHT, which directly drives formation of assemblies containing multiple HVEM molecules, monomeric BTLA and CD160 may activate HVEM receptor to promote NF-κB signaling and cell survival (Cheung et al., 2009a; Cheung et al., 2009b) through other mechanisms. The membrane-anchored forms of BTLA and CD160 could drive the localized enrichment of HVEM at cell–cell interfaces and as a consequence enhance the local concentration of HVEM cytoplasmic domains and associated signaling molecules. Additionally, soluble trimeric LIGHT could contribute by driving the formation of assemblies that bring up to three molecules of HVEM into close proximity, which may facilitate increased local density of HVEM–BTLA and HVEM–CD160 complexes. The recognition interfaces in the ternary hHVEM–hLIGHT–hCD160 complex are similar to those in the binary hHVEM–hCD160 and hHVEM–hLIGHT complexes, suggesting that little molecular accommodation is required for HVEM to simultaneously engage two types of binding partners. It remains to be determined under which conditions HVEM concurrently binds LIGHT and one of its IgSF ligands, if a trimeric HVEM–LIGHT complex can contain mixed IgSF binding partners (both CD160 and BTLA), and, importantly, whether these interactions enhance BTLA- or CD160-mediated signals. Furthermore, LIGHT can be expressed in membrane-bound or soluble forms, and it is not known if the membrane-bound form also can bind HVEM simultaneously with BTLA or CD160. Previously, it was suggested that when LIGHT and BTLA are presented on the same cell membrane, membrane LIGHT might limit BTLA binding in *trans* due to steric incompatibilities associated with the position of the LIGHT and IgSF binding sites on HVEM relative to the cell membrane (Steinberg et al., 2011). In humans, the stalk region of LIGHT is 35 amino acids, while for BTLA, it is only 24 amino acids. For hCD160, it is 17 amino acids for the glycosylphosphatidylinositol (GPI)–linked form and 19 amino acids for the transmembrane form. These constraints would position BTLA and CD160 too close to the cell membrane to bind HVEM together with LIGHT (Fig. S5). Therefore, it is possible that the membrane-bound and secreted forms of LIGHT could have different impacts on HVEM–BTLA and HVEM–CD160 binding, based on their position relative to the cell membrane, but additional in vitro and in vivo studies will be required to verify this.

Whole-body and cell type–specific gene knockouts have provided important insights into the function of HVEM and its binding partners (Mintz et al., 2019; Seo et al., 2018). Elimination of expression of one member of this network, however, could have complex effects on others. For example, deletion of LIGHT not only eliminates LIGHT–HVEM interaction but also the LIGHT–LTβR interaction. It is also possible that LIGHT deletion might provide more LTβR available for binding to LTαβ_2_, and in humans, blockade of LIGHT may alter the degree of inhibition of TL1A and FasL by DcR3, a decoy receptor not present in mice. Although analysis of no single mutation can discriminate among all these possibilities, we set out to test the importance in vivo of pairwise interactions in the HVEM network in a context in which expression of all of the proteins was maintained. To do this, we mutated solvent accessible amino acids in mHVEM that are close to the ligand binding interfaces defined by structural analyses. We succeeded in identifying mHVEM muteins with selective binding in vitro for either LIGHT or for the two IgSF ligands. These HVEM proteins were expressed at normal amounts on cells in genetically altered mouse strains and were tested in vivo following oral infection with *Y. enterocolitica* and following injection with αGalCer to activate iNKT cells to cause liver inflammation. These data demonstrate a high degree of ligand selectivity in this more complete network. Our data show that LIGHT–HVEM interactions are required for host defense against *Y. enterocolitica*. In mice that retain normal expression of LIGHT and HVEM, but in which only the ability of these proteins to interact was greatly diminished, bacteria spread and weight loss were increased and survival was diminished. The phenotype was similar to mice deficient for HVEM in both T cells and ILC3s, or in whole-body knockout mice lacking LIGHT expression. There was no effect on the host response in mice in which HVEM binding to CD160 and BTLA was diminished. Similarly, liver inflammation was dependent on CD160 and/or BTLA interacting with HVEM. As suggested by other studies (Iwata et al., 2010; Kim et al., 2019; Miller et al., 2009), this behavior may be due to the loss of inhibitory signaling in the iNKT cells that initiate this inflammatory response. It was not greatly dependent on LIGHT binding to HVEM, suggesting LIGHT-induced HVEM trimerization is not a major factor in promoting or inhibiting BTLA and CD160 signaling in this system.

It is not known why individual HVEM ligands are important for mediating biological effects in particular contexts and how the great difference in binding affinity between LIGHT and the IgSF binding partners contribute to these processes. All of the ligands activate NF-κB proteins (Cheung et al., 2009b), and there is no evidence that they employ different mechanisms for signaling through HVEM. Tissue context is likely critical in some cases. For example, it is not surprising that intestine epithelial HVEM interacts mainly with CD160 expressed by intraepithelial lymphocytes, because these cells are in continual contact with the epithelium (Shui et al., 2012), and CD160 is the only HVEM binding partner intraepithelial lymphocytes highly express. Reverse signaling by HVEM through either CD160 or BTLA could drive the biology in other instances, as reported recently for the germinal center response (Mintz et al., 2019) or in the liver inflammation model (Iwata et al., 2010; Kim et al., 2019; Miller et al., 2009). Ultimately, a deeper understanding of the biological effects of HVEM may permit the safer use of muteins and other reagents in a therapeutic context, such as in cancer immunotherapy, where soluble HVEM has shown benefit in a mouse model of lymphoma (Pasero and Olive, 2013; Šedý and Ramezani-Rad, 2019), or for treating inflammatory diseases.

## Materials and methods

### Molecular cloning and mutagenesis

A portion of the hHVEM gene encoding residues L39-C162 and mHVEM encoding residues Q39-T142 were amplified by PCR, and the resulting DNA fragments were digested with endonucleases BglII and AgeI and ligated into plasmid pMT/BiP/V5-His for His-tag fusion protein production in *Drosophila* S2 cells. DNA fragment encoding the amino acid sequence “HHHHHHG” fused to hLIGHT (L83-V240) was cloned into pMT/BiP/V5-His. The mCD160 gene encoding residues 30I-154H with the C terminus fused with amino acids “HHHHHHGGGGSGLNDIFEAQKIEWHE” was cloned into pET3a. The DNA sequences encoding a protein biological composed of mHVEM residues (Q39-Q206) followed by human IgG1 and a subsequent hexa-His tag sequences were cloned into pcDNA 3.3 vector (Life Technologies) using In-fusion HD cloning enzyme premix (Clontech). DNA fragment encoding the amino acid sequence “HHHHHHGG” fused to the N-terminus of the single chain homotrimeric mLIGHT extracellular domain (G73-V239) connecting by two (GGGGS)_4_ linkers was cloned into pcDNA 3.3 vector (Life Technologies).

A DNA fragment encoding residues of L39-V202 of hHVEM was cloned into an engineered pEGFP-N1 vector (Clontech) for expression as a protein fused with a PD-L1 transmembrane domain followed by the fluorophore enhanced GFP (EGFP) at the C terminus. The hHVEM mutant library was generated using the QuickChange II Site-Directed Mutagenesis Kit (Agilent Technologies). Full-length WT mHVEM and mutants were cloned into pmCherry-N1 vector (Clontech), respectively. Full-length mBTLA was cloned into pEGFP-N1 vector (Clontech). Full-length mLIGHT was cloned into pIRES2-EGFP vector (Clontech), which contains a subsequent IRES (internal ribosome entry site) sequence following by a fluorescent EGFP ORF. For the in vitro HVEM signaling assay, full-length proteins, including mHVEM^WT^, mHVEM^−BT/160^, mHVEM^−LIGHT^, mCD160, and mLIGHT, were cloned into pEGFP-N2 vector (Clontech) with a stop codon so they were not expressed as fusion proteins with EGFP.

### Protein production and purification

All hHVEM, hLIGHT, and mHVEM proteins were expressed and purified as previously described (Liu et al., 2015). The extracellular domains of hHVEM (L39-C162), hLIGHT (L83-V240), and mHVEM (Q39-T142) were separately cloned into the pMT/BiP/V5-His A vector (Invitrogen) and cotransfected into *Drosophila* S2 cells with the pCoBlast (Invitrogen) plasmid at a 20:1 ratio. A stable cell line was selected with blasticidin following the manufacturer’s protocol (Invitrogen). All hHVEM, hLIGHT, and mHVEM expression were induced with copper sulfate (500 µM final concentration). The proteins from filtered culture supernatants were purified by nickel-nitrilotriacetic acid column (QIAGEN) and SEC (HiLoad Superdex 75; Amersham). The single-chain hCD160-hHVEM fusion protein was expressed in *Drosophila* S2 cells and purified to homogeneity as previously described (Liu et al., 2019). The mCD160 protein was purified as inclusion bodies and refolded as previously described (Liu et al., 2019). The expression vectors encoding mHVEM (Q39-Q206) fused with human IgG1 and a subsequent hexa-His tag sequences were transfected into Expi293 (Gibco) cells using the ExpiFectamine 293 transfection kit (Gibco), and the resulting proteins were purified using Ni-resins (Qiagen). The vector encoding a hexa-His tag fused to a single chain homotrimeric mLIGHT extracellular domain (G73-V239) connecting by two (GGGGS)_4_ linkers was transfected into Expi293 (Gibco) cells using the ExpiFectamine 293 transfection kit (Gibco) and the resulting proteins were purified using Ni-resins (Qiagen) and SEC (HiLoad Superdex 75; Amersham). The resulting purified mLIGHT proteins were used immediately for experiments.

### Cell culture

Transformed *Escherichia coli* cells were cultured in lysogeny broth medium supplemented with 100 mg/liter carbenicillin at 37°C. Transfected *Drosophila* S2 cells were cultured in complete Schneider’s *Drosophila* medium (Life Technologies) supplemented with 10% heat-inactivated FBS in the presence of 25 mg/liter blasticidin for establishing stable cell lines. Protein expression in *Drosophila* S2 cell lines was induced in Express Five SFM medium (Life Technologies) in the presence of 500 mM CuSO_4_ at 25°C. Expi293 or 293T cells were maintained in DMEM (Corning) with 10% FBS at 37°C with 5% CO_2_. The transfected Expi293 cells were cultured at 37°C with 5% CO_2_ for flow cytometry analysis or at 30°C with 5% CO_2_ for protein expression.

### Crystallization, structure determination, and refinement

The purified hHVEM and hLIGHT proteins were concentrated separately and mixed in a 1:1 molar ratio to generate the hHVEM–hLIGHT complex at a concentration of 3 mg/ml in 10 mM Hepes, pH 7.0, and 150 mM NaCl solution. The resulting hHVEM–hLIGHT complex was crystallized by sitting drop vapor diffusion using 0.5 µl protein and 0.5 µl precipitant composed of 0.1 M Bis-Tris, pH 5.5, 0.2 M MgCl_2_, and 9% PEG3350. Crystals were cryoprotected by immersion in crystallization buffer supplemented with 20% of glycerol and flash-cooled in liquid nitrogen. The purified single-chain hCD160–hHVEM proteins and hLIGHT were concentrated separately and mixed in a 1:1 molar ratio to generate the hHVEM–hLIGHT –hCD160 complex at a concentration of 5 mg/ml in 10 mM Hepes, pH 7.0, and 150 mM NaCl solution. The resulting hHVEM–hLIGHT–hCD160 complex was crystallized by sitting drop vapor diffusion using 0.5 µl of protein and 0.5 µl of precipitant composed of 12% (wt/vol) PEG3350 and 4% (vol/vol) tacsimate. Crystals were cryoprotected by immersion in crystallization buffer supplemented with 20% ethylene glycerol and flash-cooled in liquid nitrogen. The purified mHVEM was concentrated to 3 mg/ml in 10 mM Hepes, pH 7.0, and 150 mM NaCl solution and then crystallized by sitting drop vapor diffusion using 0.5 µl protein and 0.5 µl precipitant composed of 90% (vol/vol) solution A with 0.2 M lithium sulfate, 0.1 M sodium acetate/acetic acid, pH 4.5, 30% (wt/vol) PEG 8000, and 10% (vol/vol) solution B with NDSB-211. Crystals were cryoprotected by immersion in crystallization buffer supplemented with 40% of glycerol and flash-cooled in liquid nitrogen.

Diffraction data from the hHVEM–hLIGHT complex were collected at Brookhaven National Laboratory beamline X29 (Table 1). Diffraction data from hHVEM–hLIGHT–hCD160 complex and mHVEM were collected at Advanced Photon Source Sector 31, Argonne National Laboratory (Table 1). All diffraction data were integrated and scaled with HKL2000 (Otwinowski and Minor, 1997). Phases of the hHVEM–hLIGHT complex were calculated by molecular replacement using the existing PDB structures 4KG8 and 4FHQ as the starting models and the software Molrep in the CCP4 package (Winn et al., 2011). Phases of hHVEM–hLIGHT–hCD160 complex were calculated by molecular replacement using the existing PDB structure 6NG9 and hHVEM–hLIGHT complex (PDB entry 4RSU) as the starting models and the software Molrep in the CCP4 package (Winn et al., 2011). Phases of mHVEM were calculated by molecular replacement using the existing PDB structure 4FHQ as the starting model and the software Molrep in the CCP4 package (Winn et al., 2011). Electron density maps were manually inspected and improved using COOT (Emsley et al., 2010). Following several cycles of manual building in COOT and refinement in REFMAC5, the hHVEM–hLIGHT complex R_work_ and R_free_ converged to 18.4% and 22.6%, respectively (Emsley et al., 2010; Winn et al., 2011).

### Mutagenesis screening

500 ng WT and mutants of hHVEM-GFP fusion plasmids in 50 µl PBS were mixed with 50 µl of 0.04 M polyethyleneimine, respectively. The mixtures were kept still for 10 min and then added separately to a 24-well plate with each well containing 1 ml of 10^6^/ml HEK293-Freestyle cells (Invitrogen). The transfected cells were cultured by shaking at a speed of 200 rpm at 37°C for 72 h followed the transfection, and then the cells were collected and resuspended in PBS. Cells from each well were further diluted to 10^6^ cells/ml.

100 µl of the diluted transfected cells was incubated separately with hCD160-6×His tag, hBTLA-6×His tag (R&D Systems), and hLIGHT-6×His tag proteins (made by the methods described above) in the mixtures with anti-6×His tag PE-labeled antibody (Abcam) for 20 min on ice. The cells were subsequently spun down, washed once, and resuspended in 100 µl PBS buffer containing additional 0.5% BSA and then subjected to flow cytometric analysis. The cells were gated on GFP-positive cells to ensure hHVEM expression and analyzed for the percentage of PE-positive cells. The binding of WT hHVEM was normalized as 1. The relative binding of hHVEM mutants were calculated by comparing the PE-positive cell percentage to the control WT hHVEM groups. The error bars reflect the results of three independent experiments.

The mHVEM, mBTLA, and mLIGHT constructs were transfected into HEK293 FreeStyle (Life Technologies) cells using polyethyleneimine (linear polyethyleneimine with a molecular weight of 25,000; Polysciences). After ∼2–3 d, the cells were harvested and diluted to 10^6^/ml. For measuring cell–cell interactions, 100 µl of cells expressing mHVEM-mCherry proteins was mixed with 100 µl of cells expressing mBTLA-EGFP or mLIGHT-IRES-EGFP proteins and then subjected to shaking at 900 rpm using a bench top microplate shaker (catalog no. 12620-928; VWR) at room temperature for 2 h. These cells were further recorded and analyzed by flow cytometry. For protein staining, 100 µl of cells expressing mHVEM-mCherry proteins was mixed with 0.3 µg mBTLA-penta-His-tag/mCD160-biotin proteins and 0.5 µg of green fluorescent anti-His-tag (catalog no. ab1206; Abcam)/Alexa Fluor 488–conjugated streptavidin (catalog no. S11223; Life Technologies) proteins. The cells were incubated for 30 min with shaking at room temperature and washed once by PBS containing 0.2% BSA (PBS-BSA). The cells were resuspended in 100 µl of PBS-BSA and analyzed by flow cytometry.

### Octet biolayer interferometry

For measuring binding affinities, mHVEM-hIgG1 was immobilized on the sensors (ForteBio) and then challenged with different concentrations of mLIGHT, mBTLA, or mCD160. The results were exported and then analyzed using Prism 5 (GraphPad Software). Final response curves were generated after subtracting the responses of the control groups. The equilibrium dissociation constants (K_D_) of the mHVEM-hIgG1 interaction with mLIGHT were calculated based on the response curves by fitting the data to the equation Y = B_max_ X/(X + K_D_), where Y is the averaged maximum response of each experiment, X is the concentration of the analytes, and B_max_ is the maximum specific binding. The equilibrium dissociation constants (K_D_) of mHVEM–hIgG1 interaction with mBTLA or mCD160 were calculated based on the 1:1 Langmuir model.

### HVEM signaling assay

Plasmid pGL4.32[luc2P/NF-κB-RE/Hygro] (NF-κB–driven firefly luciferase; Promega) and pRL-TK (*Renilla* luciferase as an internal control; Promega) were cotransfected with mHVEM^WT^, mHVEM^−BT/160^, mHVEM^−LIGHT^, or control (vector only) into 293T cells by TransIT-LT1 Transfection Reagent (Mirus). 24 h later, transfected cells were co-cultured with control, mCD160-, or mLIGHT-transfected 293T cells. Luciferase activity was measured on the EnVision 2104 Multimode Plate Reader (PerkinElmer) using the Dual-Glo Luciferase Assay System kit (Promega) after another 18 h.

### Generation of mHVEM mutant mice

The mHVEM mutant mice were generated using the CRISPR-Cas9 system. The transgenic mouse core of the University of California (UC), San Diego Moores Cancer Center injected the single guide RNA (sgRNA)–Cas9 complex plus a specific single-stranded DNA homology-directed repair (HDR) template into C57BL/6 pronuclear embryos. All materials of the CRISPR-Cas9 system were designed and ordered from Integrated DNA Technologies. Two specific sgRNAs targeted exon 3 of the *Tnfrsf14* locus: sgRNA-1 for *Tnfrsf14*^G72R/V74A^ (mHVEM^−BT/160^; 5′-CAG​GTC​TGC​AGT​GAG​CAT​AC-3′) and sgRNA-2 for *Tnfrsf14*^H86D/L90A^ (mHVEM^−LIGHT^; 5′-ACA​TAT​ACC​GCC​CAT​GCA​AA-3′) Two specific single-stranded DNAs were used as HDR templates: mHVEM^−BT/160^ (5′-TGG​CTG​CAG​GTT​ACC​ATG​TGA​AGC​AGG​TCT​GCA​GTG​AGC​ACA​CGC​GTA​CAG​CGT​GTG​CCC​CCT​GTC​CCC​CAC​AGA​CAT​ATA​CCG​CCC​ATG​CA-3′) and mHVEM^−LIGHT^ (5′-CAG​GCA​CAG​TGT​GTG​CCC​CCT​GTC​CCC​CAC​AGA​CAT​ATA​CAG​CGG​ACG​CTA​ATG​GCG​CTA​GCA​AGT​GTC​TGC​CCT​GCG​GAG​TCT​GTG​ATC​CAG​GTA​GGA-3′). For screening, we created a new restriction enzyme site near the protospacer adjacent motif sequence, which did not alter the amino acid sequence. A new MluI or NheI site was thereby created in the KI genomes of the mHVEM^−BT/160^ or mHVEM^−LIGHT^ mice, respectively. The F0 founder pups were screened for exon 3 of the *Tnfrsf14* locus by enzyme digestion and PCR using the primers Hvem-exon3-F1 (5′-GTA​CAG​TGT​TCA​GTT​CAG​GGA​TAG-3′) and Hvem-exon3-R1 (5′-AGC​AGG​AAA​GAA​CCT​CTC​ATT​AC-3′). The *Tnfrsf14* exon 3 sequences were cloned and sequenced from each line of founder mice that had undergone HDR. The successful HDR F0 founders were first backcrossed to the WT C57BL/6 strain. Germline transmission of each line of mHVEM mutant mice (N1) was verified by PCR and restriction enzyme digestion analysis. Testing for potential off-target genes, analyzed by the software from Integrated DNA Technologies, and homologous sequences were confirmed by PCR using a specific pair of primers on each gene and sequencing at the N1 generation. We examined six potential off-target genes from mHVEM^−BT/160^ strain and four genes from mHVEM^−LIGHT^ strain. Two and four founders from mHVEM^−BT/160^ or mHVEM^−LIGHT^ strain, respectively, were verified and backcrossed again to the WT C57BL/6 mice. After two backcrosses with C57BL/6 mice, we obtained heterozygous (KI/+) mice (N2) from each mHVEM mutant strain. We obtained homozygous offspring (N2F1) by intercrossing the N2 generation of KI/+ mice. Age- and gender-matched co-housed littermates were used for experiments. All mice were bred and housed under specific pathogen–free conditions in the vivarium of La Jolla Institute for Immunology (LJI) and all animal experimental procedures were approved by the LJI Animal Care and Use Committee.

### Bacterial infection

*Y. enterocolitica* strain WA-C (pYV::CM) was prepared as described previously (Seo et al., 2018; Trülzsch et al., 2004). Briefly, *Yersinia* were grown overnight in lysogeny broth at 30°C, and the overnight culture was expanded with fresh medium for 6 h. Bacteria were washed and diluted with PBS. Co-housed male littermates were infected by oral gavage with 1 × 10^8^ CFU *Y. enterocolitica*. Infected mice were analyzed by measurement of body weight daily for up to 12 d. Tissues were harvested at up to 7 d after infection or at the time of death for histopathology analysis as described previously (Seo et al., 2018). Mice were considered deceased if they had lost 30% of their body weight and immediate euthanasia was required. In the weight loss experiments, the body weights of the dead mice were carried forward.

### Histopathology analysis

We harvested several spleen and liver samples from each strain which had representative symptoms at 7 d after *Y. enterocolitica* infection, fixed in zinc formalin, routinely embedded into paraffin blocks, cut at 4 µm thickness, and stained with either H&E or the Warthin–Starry (WS) silver method. Slides were scanned with a Zeiss Axio Scan.Z1 Digital Slide Scanner. Splenic and hepatic H&E sections were blinded to conditions and provided with both glass slides and whole slide images to a board-certified pathologist familiar with *Yersinia*-induced disease. After reviewing all slides, the pathologist determined there were six categories of lesions in each the liver and spleen. For splenic slides: 0, white pulp lymphoid hyperplasia, no *Yersinia* colonies; 1, focal marginal zone necrosis, no *Yersinia* colonies; 2, multifocal marginal zone necrosis, no *Yersinia* colonies; 3, large marginal zone *Yersinia* colonies with no necrosis/inflammation; 4, multifocal marginal zone *Yersinia* colonies with some necrosis or splenitis; 5, large marginal zone *Yersinia* colonies with necrotizing splenitis; 6, large marginal zone *Yersinia* colonies with abscesses. For hepatic slides: 0, no lesions; 1, minimal hepatitis or hypercellularity, no *Yersinia* colonies; 2, mild multifocal necrotizing hepatitis, no *Yersinia* colonies; 3, moderate multifocal necrotizing hepatitis, no *Yersinia* colonies; 4, multiple *Yersinia* colonies, no inflammation; 5, moderate necrotizing hepatitis with *Yersinia* colonies; 6, marked necrotizing hepatitis with *Yersinia* colonies. Slides were then reviewed again and assigned a score.

### Hepatic injury

Co-housed female littermates were inoculated with 2 µg αGalCer (KRN7000; Kyowa Kirin Research) in a total volume of 200 µl PBS by retro-orbital injection. Serum ALT activity was measured using a colorimetric/fluorometric assay kit (K752; Biovision) at 16 or 24 h after injection. Hepatic tissues were collected, and the necrotic areas were determined using H&E staining at 24 h after αGalCer treatment.

### Statistical analysis

All data were randomly collected and analyzed using Microsoft Office Excel and GraphPad Prism 9 software. Data are shown as mean with the SEM or SD. Details regarding the statistical analysis and the representative number of mice (*n*) are indicated in each figure legend. Statistical significance is indicated as follows: * P < 0.05; **, P < 0.01; ***, P < 0.001; ****, P < 0.0001.

### Online supplemental material

Fig. S1 shows the binding interface between hHVEM and hLIGHT. Fig. S2 shows the relative binding affinities of the HVEM mutants with BTLA, CD160, and LIGHT and selective signaling by mHVEM muteins. Fig. S3 shows the outcome of CRISPR-Cas9 editing of exon 3 of the *Tnfrsf14* locus and that mHVEM^-BT/160^ and mHVEM^−LIGHT^ mice have normal surface HVEM expression. Fig. S4 shows the outcome of *Y. enterocolitica* infection in mHVEM^−BT/160^ mice. Fig. S5 illustrates a model for the stalk regions of BTLA, CD160, and LIGHT.
